# Interactions of low-energy electrons with the FEBID precursor chromium hexacarbonyl (Cr(CO)_6_)

**DOI:** 10.3762/bjnano.8.258

**Published:** 2017-12-04

**Authors:** Jusuf M Khreis, João Ameixa, Filipe Ferreira da Silva, Stephan Denifl

**Affiliations:** 1 Institut für Ionenphysik und Angewandte Physik, Leopold Franzens Universität Innsbruck, Technikerstrasse 25, A-6020 Innsbruck, Austria; 2 Laboratório de Colisões Atómicas e Moleculares, CEFITEC, Departamento de Física, Faculdade de Ciências e Tecnologia, Universidade NOVA de Lisboa, Campus de Caparica, 2829-516 Caparica, Portugal

**Keywords:** chromium hexacarbonyl Cr(CO)_6_, dissociative electron attachment, electron ionization, FEBID, metastable decay

## Abstract

Interactions of low-energy electrons with the FEBID precursor Cr(CO)_6_ have been investigated in a crossed electron–molecular beam setup coupled with a double focusing mass spectrometer with reverse geometry. Dissociative electron attachment leads to the formation of a series of anions by the loss of CO ligand units. The bare chromium anion is formed by electron capture at an electron energy of about 9 eV. Metastable decays of Cr(CO)_5_^−^ into Cr(CO)_4_^−^, Cr(CO)_4_^−^ into Cr(CO)_3_^−^ and Cr(CO)_3_^−^ into Cr(CO)_2_^−^ are discussed. Electron-induced dissociation at 70 eV impact energy was found to be in agreement with previous studies. A series of Cr(CO)*_n_*C^+^ (0 ≤ *n* ≤ 3) cations formed by C–O cleavage is described for the first time. The metastable decay of Cr(CO)_6_^+^ into Cr(CO)_5_^+^ and collision-induced dissociation leading to bare Cr^+^, are discussed. In addition, doubly charged cations were identified and the ration between doubly and singly charged fragments was determined and compared with previous studies, showing considerable differences.

## Introduction

Organometallic compounds have been extensively studied since they are used for a broad field of applications. Among the variety of applications, nanotechnologies have caught special attention since organometallic compounds can be used as a precursor to deposit metals on a surface. The conventional lithography techniques are approaching the limits of spatial resolution [1], therefore it is crucial to search and improve new methods and techniques for future technological requirements. Focused electron beam induced deposition (FEBID) can be considered an assisted chemical vapour deposition (CVD) technique. However, in the former case the organometallic precursor is not fragmented by thermal energy but instead by a high-energy electron beam. The precursor molecules are delivered to the substrate in the gas phase and further irradiated by a high-energy electron beam. The electron beam decomposes the precursor molecules, leaving the metal on the surface and the organic ligands are pumped away [2–3]. FEBID has shown high potential in growing defined three-dimensional structures close to any geometry and to write on uneven surfaces. Although FEBID is a promising technique, improvements are still needed in order to get pure and highly resolved deposits. CVD precursors are normally used as FEBID precursors; however, their performance is limited, leading to co-deposition of ligands and ligand fragments together with the desired metal, with the formation of non-defined deposits on the surface.

When high-energy electrons interact with the surface, a cascade of low-energy electrons (LEE) and backscattered electrons are generated. Many chemical reactions can be triggered by those secondary electrons with an energy distribution characterised by a substantial fraction close to the ionization energy of FEBID precursors, peaking well below 10 eV and extending with appreciable intensities down to 0 eV [4]. The quality of the formed nanostructures is controlled and influenced by the interactions of the secondary and backscattered electrons with the precursor molecules. LEE initiate chemical reactions on the surface by dissociative electron attachment (DEA) and dissociative electron ionization, as well as neutral dissociation. Those processes need to be well understood, in order to maximise the quality of deposited metal as well as to minimise the adverse or unwanted effects, such as non-pure metal deposition resulting from the co-deposition of ligands.

In order to improve the quality of metallic deposits, LEE interactions with organometallic precursors have been studied. Several studies have been reported, e.g., DEA studies with η-(C_3_H_5_)Ru(CO)_3_Br [5] and the bimetallic precursor HFeCo_3_(CO)_12_ [6–7]. Results of electron interactions with platinum-based precursors, such as Pt(PF_3_)_4_ and MeCpPtMe_3_ [8–10], as well as with Co(CO)_3_NO and W(CO)_6_ [11–13] were also reported. Metal carbonyl precursors were investigated by electron transmission spectroscopy describing the negative ion states [14], and electron attachment thresholds for Cr(CO)_6_, Mo(CO)_6_ and W(CO)_6_ were reported [15]. Electron attachment to tungsten hexacarbonyl [13] and tungsten hexachloride [16], as well as electron ionization studies with those molecules were performed with proposed fragmentation pathways and determined threshold energies for the formed cationic species [17]. Electronic energy levels of metal carbonyls and metal cyanides were described by Gray and Beach [18], and the molecular orbitals were calculated by Johnson and Klemperer using the SCF-Xα-MSW method [19]. Negative ions were previously reported for a series of pentacarbonyl metals of group VI in the periodic table including Fe(CO)_5_, showing dissociation by capture of LEE with an energy close to 0 eV [20]. Electron ionization of W(CO)_6_ clusters was also recently investigated [21] showing the sequential decay of the ionized organometallic precursor. Aggregates of Fe(CO)_5_ deposit on Ar nanoparticles were studied by Lengyel and co-workers [22]. In this study strong differences in electron-induced decomposition of aggregates of iron pentacarbonyl (Fe(CO)_5_) when compared to electron attachment under isolated conditions were observed. The ion yield curves (ion yield plotted as a function of the initial electron energy) for the formation of cluster anions containing two or more iron atoms turned out to be different from those of Fe(CO)_5_ in the gas phase.

The dimer metal cation W_2_^+^ resulting from electron ionization of the neutral W(CO)_6_ dimer was reported by Neustetter et al. [23] showing the fast conversion of the weak cluster bond into a strong covalent metallic bond. In comparison to electron collisions, anisotropic coulombic explosion of CO ligands upon multiple ionization in a femtosecond laser field was recently reported by Tanaka et al. [24] for Cr(CO)_6_, Mo(CO)_6_ and W(CO)_6_.

In the present work we studied the interaction of LEE with the chromium carbonyl precursor, Cr(CO)_6_. In general, a low-energy electron may be captured by a target molecule, which leads to the formation of a transient negative ion (TNI). This electronically and/or vibrationally excited TNI will relax by formation of a negatively charged fragment ion and neutral fragment(s) (DEA) or via electron detachment leaving the target molecule eventually in an excited state. If the incident electron energy is higher than the ionization threshold of the molecule, electron ionization and dissociation is energetically possible. The formation of both positive and negative ions, from the molecular ion as well as from intermediate ions formed, may also occur in a slow reaction extending to the microsecond-timescale (metastable decay).

## Results and Discussion

### Dissociative electron attachment

**Figure 1:**
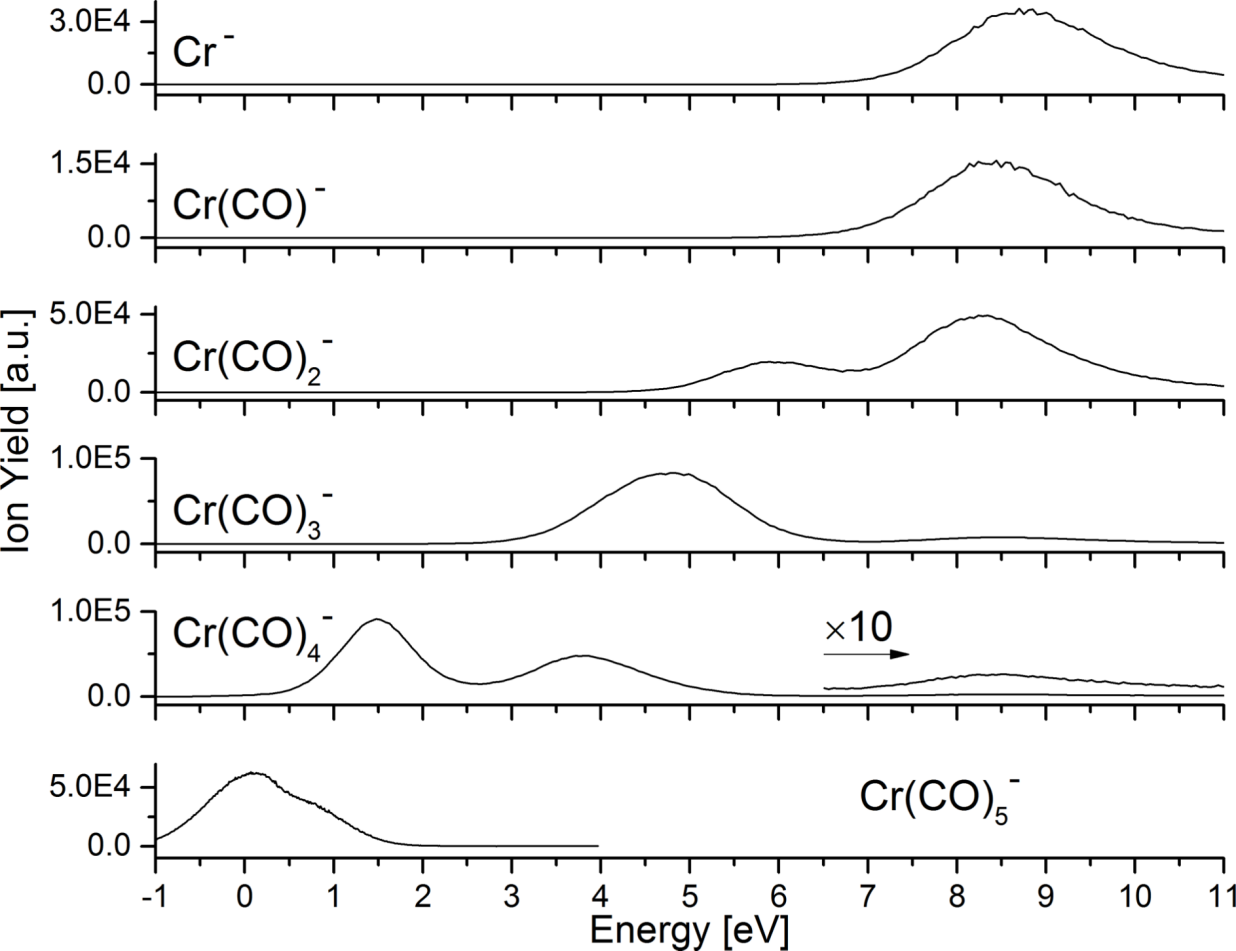
Ion yield curves of negatively charged fragments formed by dissociative electron attachment to chromium hexacarbonyl, Cr(CO)_6_.

Figure 1 shows the ion yield curves of the negative ions formed upon electron attachment. When the incoming electron attaches to the Cr(CO)_6_ molecule, the transient negative ion is formed, Cr(CO)_6_^#−^. The excess electron should occupy the lowest unoccupied molecular orbital (LUMO) 9a_1g_ with sigma character [19], or one of the higher-lying virtual molecular orbitals. The ion yields for the detected anionic species are in agreement with Pignataro and co-workers [20]. The dissociative channels described in this study follow the reactions below:


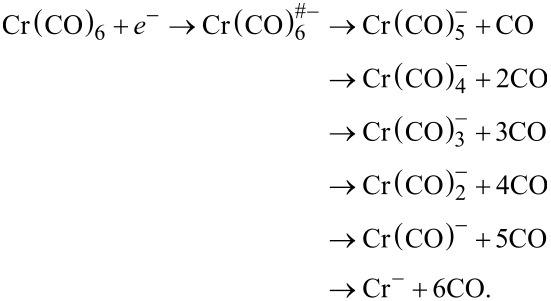


These six reactions describe the series of loss of the organic CO ligand. In contrast to W(CO)_6_ [13], the series of CO loss is complete in the present case, with the formation of the bare metal anion. This is in agreement with Pignataro et al. [20], where the six anionic species where reported. The corresponding anion yields indicate that the number of leaving CO ligands increases with the electron energy. In Table 1 appearance energies and peak positions are listed and compared with the results from Pignataro and co-workers. The appearance energies are in good agreement, and the maximum deviation from the previous values is 0.45 eV for Cr^−^ formation [20].

**Table 1:** Appearance energies and peak energies for the negative ions formed by DEA to Cr(CO)_6_.

anion	mass (u)	AE (eV)	AE (eV) [20]	peak energy (eV)^a^			peak energy (eV) [20]		

Cr(CO)_5_^−^	192	0.0	0.10	0.1	—	—	0.4	—	—
Cr(CO)_4_^−^	164	0.5	0.60	1.5	3.8	8.6	1.5	3.9	—
Cr(CO)_3_^−^	136	2.9	3.00	—	4.7	8.7	—	4.3	—
Cr(CO)_2_^−^	108	4.6	4.50	—	5.9	8.3	—	6.0	8.5
Cr(CO)^−^	80	6.2	6.00	—	—	8.5	—	—	7.9
Cr^−^	52	6.4	6.85	—	—	8.8	—	—	8.8

^a^Values are taken of the maxima of the peaks.

The Cr(CO)_5_^−^ ion is formed through resonance near 0 eV, reflected in a peak maximum of the ion yield at 0.1 eV, i.e., slightly red-shifted compared to the maximum position of 0.4 eV being reported previously. The Cr(CO)_4_^−^ ion is formed via three different resonances: two low-energy resonances in agreement with previous studies, and a third resonance, reported here for the first time, and evident through a maximum in the ion yield curves centred at 8.6 eV. The anions Cr(CO)_3_^−^, Cr(CO)_2_^−^, Cr(CO)^−^ and Cr^−^ are formed through resonances contributing to the respective ion yields above 4.0 eV. These are apparent through two maxima of the Cr(CO)_3_^−^ and Cr(CO)_2_^−^ ion yields at 4.7 eV and 8.7 eV and at 5.9 eV and 8.3 eV, respectively. In the Cr(CO)^−^ and Cr^−^ ion yields, only contributions through the higher lying resonance are visible. These appear with a maximum at 8.5 eV and 8.8 eV, respectively. In the case of Cr(CO)_3_^−^ the high-energy resonance appearing in the ion yields at 8.7 eV is reported here for the first time. The anions Cr(CO)^−^ and Cr^−^, are formed through high lying resonances with maxima in the respective ion yields at 8.5 eV and 8.8 eV.

As described below in the Experimental section, metastable decay processes were studied. By a proper tuning of the magnetic sector, it is possible to separate the metastable anion that will decay into a lighter anion and a neutral fragment. In the present studies we have observed three different metastable decays:


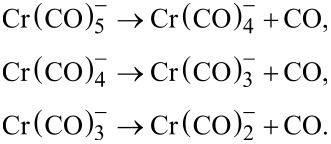


**Figure 2:**
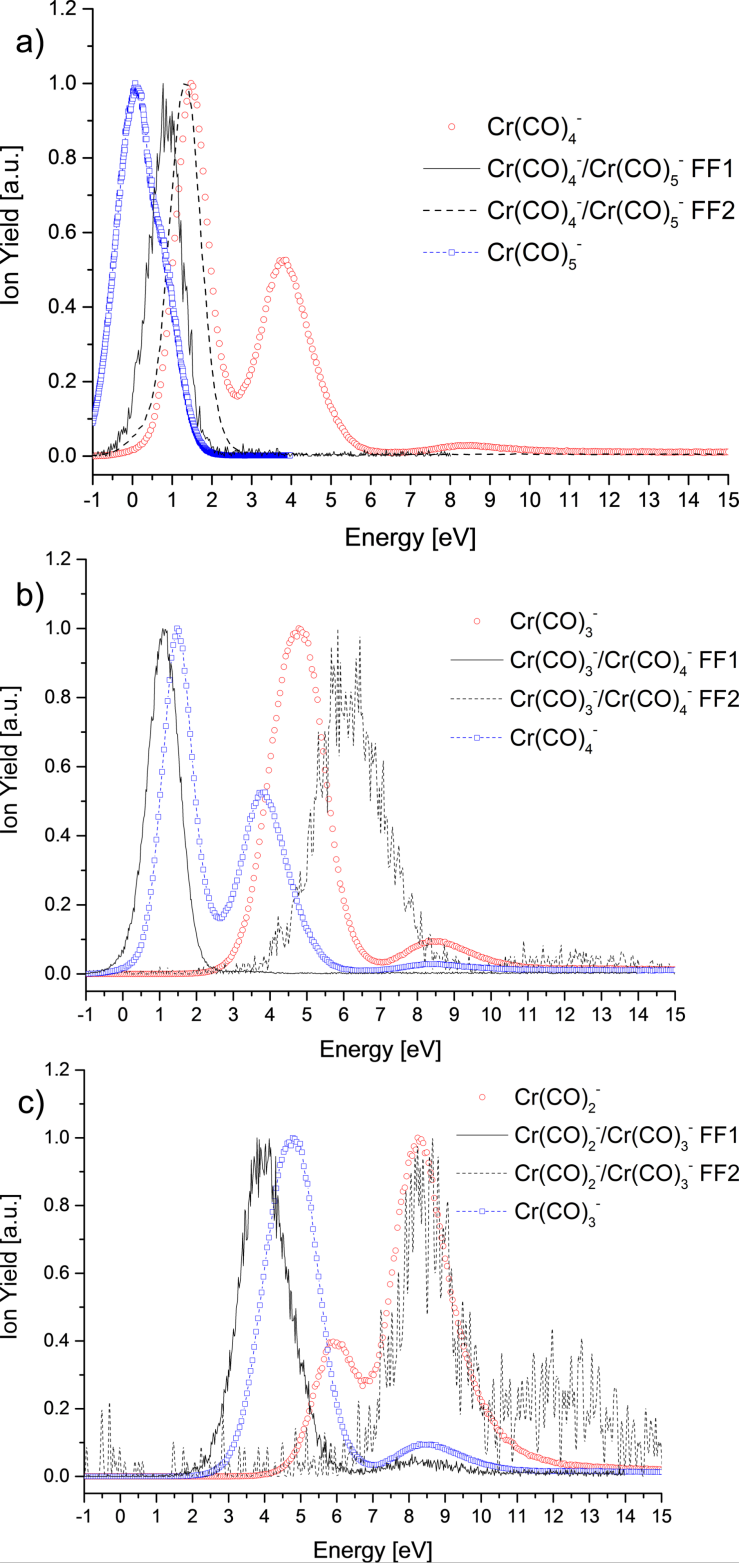
Electron energy dependence for the metastable decay reactions in the FF1 and the FF2, as well as the prompt ion yields for the involved fragment anions: a) for the reaction of Cr(CO)_5_^−^ into Cr(CO)_4_^−^, b) for the reaction of Cr(CO)_4_^−^ into Cr(CO)_3_^−^ and c) for the reaction of Cr(CO)_3_^−^ into Cr(CO)_2_^−^. Ion yields are normalized.

In Figure 2 the energy dependence for these three decay reactions is shown. Decays happening in the ion source are called prompt decays, i.e., precursor anions have a lifetime below 3–4 μs. This corresponds to the flight time till the first field-free region (FF1). In the second field-free region (FF2), the flight time varies between 16 and 20 μs, depending on the ion. Table 2 summarizes the entrance and exit times for different anions in FF1 and FF2.

**Table 2:** Flight time in FF1 and FF2 for different anions (values in µs).

anion	Cr(CO)_5_^−^		Cr(CO)_4_^−^		Cr(CO)_3_^−^		Cr(CO)_2_^−^	
	entrance	exit	entrance	exit	entrance	exit	entrance	exit

FF1	4	18	4	17	4	15	3	14
FF2	24	44	22	41	20	38	19	35

Figure 2a shows the ion yields for the metastable decay of Cr(CO)_5_^−^ into Cr(CO)_4_^−^ as well as for the prompt dissociation for Cr(CO)_5_^−^ and Cr(CO)_4_^−^. The ion yields of the metastable decay are located in between the maxima for both promptly formed anions. Johnson and Klemperer [19] described the lowest seven unoccupied orbitals, which should be related with the present metastable decays. The prompt loss of the CO ligand leading to Cr(CO)_5_^−^ is due to electron capture in a repulsive mixed σ–π state [19] LUMO located at around 0.1 eV and from another state located at 0.9 eV, which appears as shoulder in the ion yield. The decay into Cr(CO)_4_^−^ in the FF1 arises from the latter state of Cr(CO)_5_^−^, assuming that the excess of energy is distributed randomly by the vibrational degrees of freedom before the molecule fragments. The decay in the FF2 appears at ca. 1.3 eV. The Cr(CO)_4_^−^ anion from prompt decays is observed in a peak located at 1.5 eV and another peak at 3.8 eV that should be related to a different TNI state than those leading to metastable decays. The ion yield of the decay of Cr(CO)_4_^−^ into Cr(CO)_3_^−^ (Figure 2b) has an uncommon behaviour. The prompt ion yield of Cr(CO)_3_^−^ shows two peaks at 4.8 eV and 8.3 eV, which emerge through two different resonances. The first peak in the ion yield of the metastable decay in the FF1 is observed at a lower energy than the first maximum of the prompt Cr(CO)_4_^−^ yield. This may indicate that the electron is captured in an orbital that does not lead to a stable Cr(CO)_4_^−^ ion detectable in the time window of the mass spectrometer. The anions formed by decays in the FF2 have the highest abundance at 6.3 eV, lower in energy than the third peak for prompt Cr(CO)_3_^−^ formation. The formation of this ion yield can be explained in terms of the energy excess in the TNI formed as explained above for the metastable decay of Cr(CO)_5_^−^. The metastable decay of Cr(CO)_3_^−^ into Cr(CO)_2_^−^ is shown in Figure 2c. The prompt dissociation into Cr(CO)_3_^−^ is most abundant at electron energies of 4.8 eV and 8.5 eV as described above. The decay of Cr(CO)_3_^−^ in the FF1 is similar to the decay of Cr(CO)_4_^−^ into Cr(CO)_3_^−^ in the FF1. The electron is captured in a different state than the one leading to the prompt dissociation. The decay in the FF2 leading to Cr(CO)_2_^−^ (peaking at ca. 8.5 eV) may involve the same orbital like for prompt decay in Cr(CO)_2_^−^. Moreover, the peak close to 8.5 eV is common to both anionic species promptly formed in the ion source and appears in the metastable yield for both field-free regions. At higher energies, ca. 12.3 eV, another peak indicating the formation of Cr(CO)_2_^−^ in the FF2 is present. This decay comes from the excess of energy deposited in the initial TNI.

### Electron ionization

**Figure 3:**
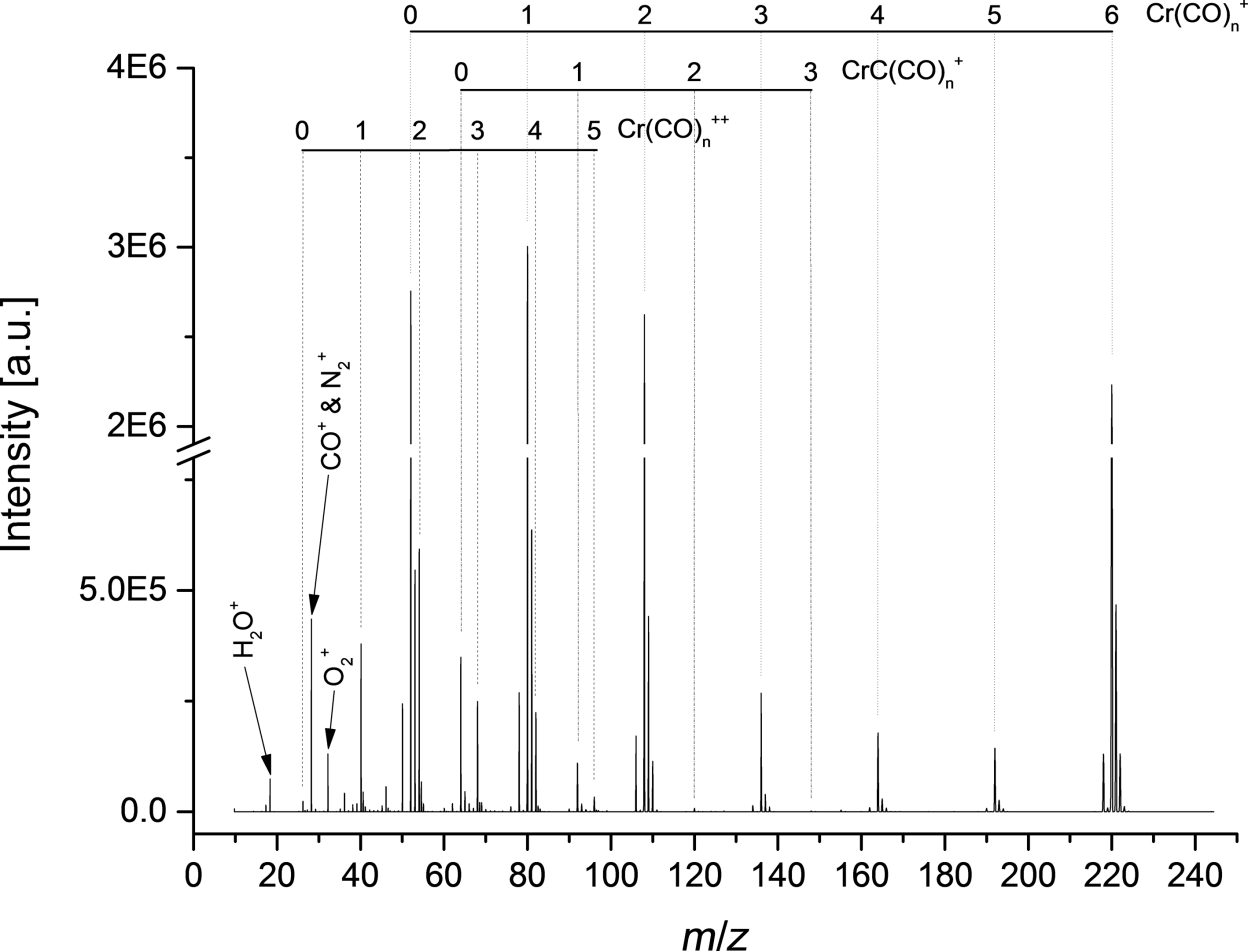
Electron ionization mass spectrum of Cr(CO)_6_ obtained at an electron energy of ca. 70 eV.

Figure 3 shows the electron ionization mass spectrum at an electron energy of ca. 70 eV. The recorded mass spectrum is in agreement with previous studies of Junk and Svec [25] and Foffani and co-workers [26]. In both studies, the most intense fragmentation channel turned out to be the formation of the bare metal cation, Cr^+^. In contrast, in the present study we observe Cr^+^ and Cr(CO)^+^ cations as preferred ions. The less intense fragment cations are, in agreement with previous studies, Cr(CO)_5_^+^, Cr(CO)_4_^+^ and Cr(CO)_3_^+^. Cr(CO)*_n_*C^+^ (0 ≤ *n* ≤ 3) is formed by the cleavage of C–O triple bonds. For this series the formation of CrC^+^ is the preferable channel, and Cr(CO)_2_C^+^ and Cr(CO)_3_C^+^ are less intense ions.

Doubly charged cations are also observed in the present study, though no signal for the doubly charged Cr(CO)_6_^2+^ was observable within the detection limit of the present apparatus. Apart from the series Cr(CO)*_n_*^+^ and Cr(CO)*_n_*^++^ (0 ≤ *n* ≤ 5), the series CrC(CO)*_n_*^+^ and CrC(CO)*_n_*^++^ (0 ≤ *n* ≤ 3) series are also observed. The intensity ratio of doubly to singly charged ions was determined from the peak maxima in the mass spectrum. The values are summarized in Table 3. When we compare our results with those from Junk and Svec [25], the doubly/singly charged ratio shows a different tendency. This difference can be attributed to the high mass resolution of the present spectrum, when compared with previous findings. In addition, it should be noted that in [25] no value of the used electron energy is stated. Different electron energies may also explain the different ratios.

**Table 3:** Ratio of doubly and singly charged fragments.

species	ratio ion^++^/ion^+^ × 100	
	present study	[25]

Cr(CO)_6_	—	1.0
Cr(CO)_5_	23.2	1.0
Cr(CO)_4_	72.9	10.0
Cr(CO)_3_	93.1	3.0
Cr(CO)_2_	19.7	13.0
Cr(CO)	12.6	—
Cr	0.9	—
CrC(CO)_3_	77.4	—
CrC(CO)_2_	97.9	—
CrC(CO)	51.5	—
CrC	0.3	—

**Figure 4:**
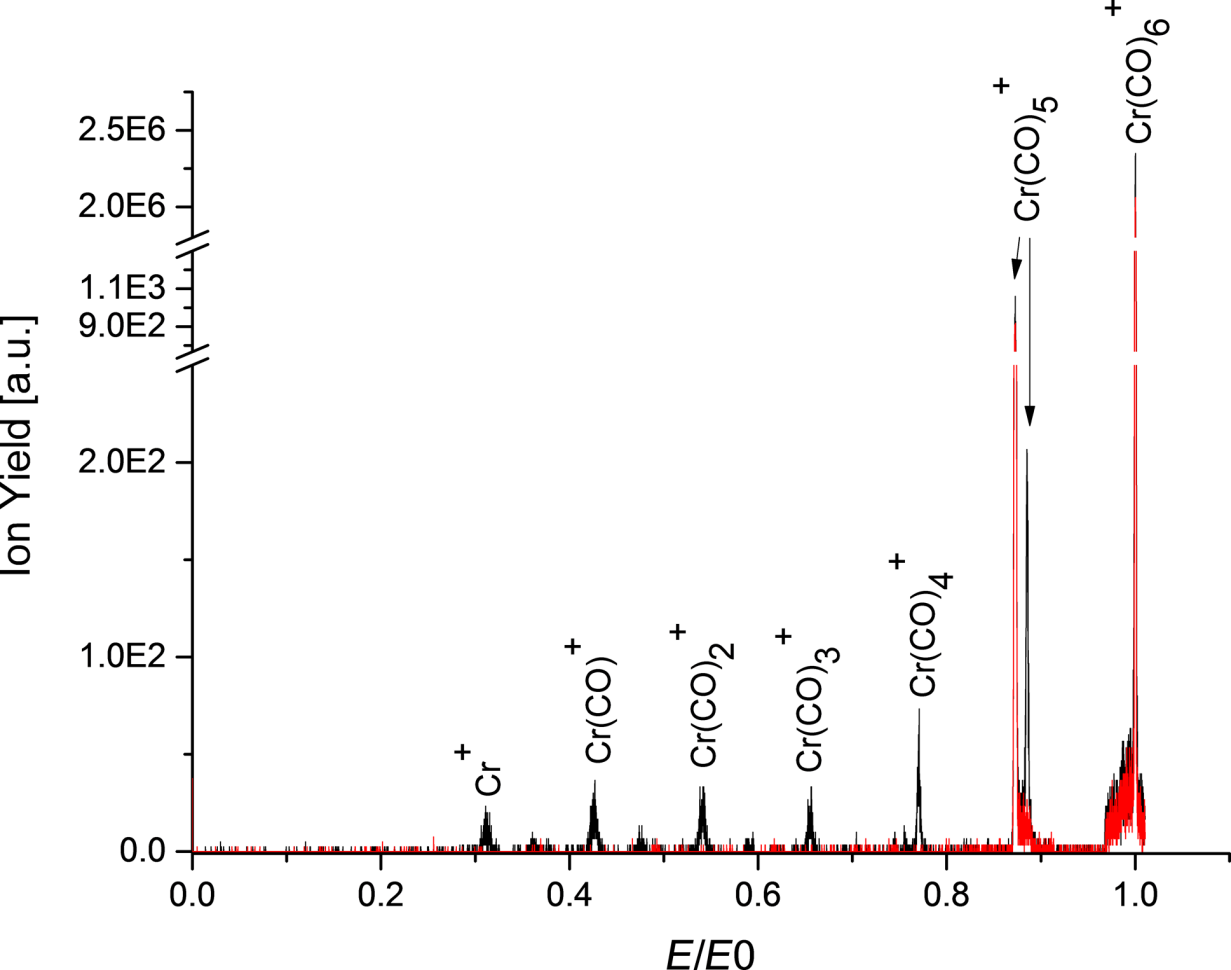
Electric sector field scan transmitting the Cr(CO)_6_^+^ precursor ion at the electric field *E*_0_ without collision gas (red curve) and with collision gas (black) in the collision cell. The fragment ions formed in the collision cell were subsequently accelerated with 600 V. Without collision gas only the metastable decay of the parent cation into Cr(CO)_5_^+^, in the FF2 was detected; in the collision-induced dissociation spectrum several more peaks appear (see text). The metastable peak is still present in this case. The initial electron energy was ca. 70 eV.

Cr(CO)_5_^+^ is also formed by the metastable decay of Cr(CO)_6_^+^ when flying through the FF2. No other metastable decays are observed for the parent ion as shown in Figure 4. In this case the metastable ion yield was detected in a scan of the electric field *E* of the electric sector after the FF2. In a subsequent experiment, collision-induced dissociation (CID) was stimulated by introducing air into a collision cell mounted in the FF2. The collision energy of Cr(CO)_6_^+^ is 677.8 ± 2.3 eV in the centre of mass system. The formed cations were subsequently accelerated by 600 V. Figure 4 shows the resulting CID spectrum of Cr(CO)_6_^+^. In CID, a complete loss of CO ligands also occurs, leading to the formation of the bare metal cation, Cr^+^. Due to the subsequent acceleration of the formed fragments in the cell, CID peaks are slightly shifted to higher electric field values, which is clearly visible for the Cr(CO)_5_^+^ peak.

## Conclusion

In this work, we have presented a detailed investigation of electron interactions with the FEBID precursor Cr(CO)_6_. Electron attachment leads to the dissociation of the compound and no molecular parent anion can be detected on mass spectrometric timescales in the present study. The most abundant anions observed in the present study were Cr(CO)_3_^−^ and Cr(CO)_4_^−^. However, we note that at electron energies close to 0 eV the electron current has not reached the regulated value of 10 μA (see section Experimental) and hence the amount of Cr(CO)_5_^−^ formed close to 0 eV may be underestimated in the present work. One common temporary negative ion state leads to the formation of fragment anions close to 8.5 eV, which is accompanied by a loss of 2–6 CO units. With the mass spectrometer utilized here, we were also able to extend the investigations for the first time to the microsecond-timescale and showed that fragment anions formed in the source may further lose a CO ligand on the way to the detector. In case of electron ionization, new fragmentation channels accompanied by C–O triple-bond cleavage are reported for the first time. The ratios of doubly to singly charged fragment ions show several differences compared to previous values, which may result from different electron energies used. On microsecond-timescales, only the metastable decay of Cr(CO)_6_^+^ into Cr(CO)_5_^+^ and CO is observable and only excess energy added through collisions induces a further loss of CO ligands.

## Experimental

Since the used experimental setup was already described in detail elsewhere [27], only a short overview will be given. A double focussing two-sector-field mass spectrometer (VG ZAB2-SEQ) in Nier–Johnson geometry was used. The ion beam was produced in a standard Nier-type ion source. The chromium hexacarbonyl (Cr(CO)_6_) sample from Sigma-Aldrich with a stated purity of >98% was filled in an external sample container. The sample container was heated to temperatures between 74 °C and 79 °C. The regulation of the electron current to 10 μA set in at an electron energy of about 2 eV. The ions were extracted by a repeller lens out of the interaction region, accelerated by a voltage drop of 6 kV, momentum-selected by the magnetic sector, energy-selected by the electric sector, and detected by a channel electron multiplier (Dr. Sjuts, Germany). Mass scans were taken by varying the magnetic field while the electric field was kept constant at electron energies of ca. 70 eV. For the study of dissociative electron attachment, the calibration of the incident electron energy scale was done by measuring the SF_6_^−^ and F^−^ resonances of SF_6_. The electron energy resolution was approximately 1 eV (FWHM) [27]. Additionally, the experimental setup allowed for the measurement of ion yields from metastable decays and collision-induced dissociation (CID). Between the acceleration region and the magnetic sector the first field-free region (FF1) is located, followed by a second field-free region (FF2) after the magnetic sector. For the observation of metastable decay processes occurring in the FF1 and the FF2 two different measurement techniques were used. As discussed by Cooks et al. [28] and by Ferreira da Silva et al. [29]*,* a metastable decay of a precursor ion (with mass *m*_p)_ into ionic fragment 1 and neutral fragment 2 (with mass *m*_f1_ and *m*_f2_) can be detected in the first field-free region by a variation of the acceleration voltage *V*_f1_. Since the fragment keeps its velocity while its kinetic energy is altered, the decay product will only pass the magnetic sector field if the acceleration voltage is increased accordingly to

[1]
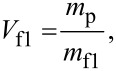


and the mass transmitted through the magnetic sector is set to

[2]
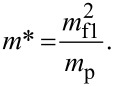


For the detection of a metastable decay in the FF2, the magnetic sector field was fixed at the mass *m*_p_ while varying the electric sector field to E* (for detection of an ion with mass *m*_f1_):

[3]
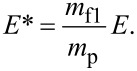


For the CID experiments a collision chamber mounted in the FF2 was used. Ambient air at variable pressures was used as a collision gas to achieve a collision-induced dissociation of the precursor ion [30]. The collision chamber allowed subsequent acceleration of the ions by applying a voltage of 600 V, which facilitated the separation of metastable and CID ion yield.

## References

[1] Hagen C W, van Dorp W F, Crozier P A, Kruit P (2008). Surf Sci.

[2] Botman A, Mulders J J L, Hagen C W (2009). Nanotechnology.

[3] Utke I, Hoffmann P, Melngailis J (2008). J Vac Sci Technol, B.

[4] Thorman R M, Kumar T. P. R, Fairbrother D H, Ingólfsson O (2015). Beilstein J Nanotechnol.

[5] Thorman R M, Bjornsson R, Ingólfsson O (2016). Eur Phys J D.

[6] Kumar T P R, Barth S, Bjornsson R, Ingólfsson O (2016). Eur Phys J D.

[7] Kumar T P R, Bjornsson R, Barth S, Ingólfsson O (2017). Chem Sci.

[8] Allan M (2011). J Chem Phys.

[9] Engmann S, Stano M, Matejčík Š, Ingólfsson O (2012). Phys Chem Chem Phys.

[10] May O, Kubala D, Allan M (2012). Phys Chem Chem Phys.

[11] Engmann S, Stano M, Matejčík Š, Ingólfsson O (2011). Angew Chem, Int Ed.

[12] Engmann S, Stano M, Papp P, Brunger M J, Matejčík Š, Ingólfsson O (2013). J Chem Phys.

[13] Wnorowski K, Stano M, Matias C, Denifl S, Barszczewska W, Matejčík Š (2012). Rapid Commun Mass Spectrom.

[14] Giordan J C, Moore J H, Tossell J A, Weber J (1981). J Am Chem Soc.

[15] Tossel J A, Moore J H, Olthoff J K (1984). J Am Chem Soc.

[16] Neustetter M, Ferreira da Silva F, Denifl S (2016). Rapid Commun Mass Spectrom.

[17] Wnorowski K, Stano M, Barszczewska W, Jówko A, Matejčík Š (2012). Int J Mass Spectrom.

[18] Gray H B, Beach N A (1963). J Am Chem Soc.

[19] Johnson J B, Klemperer W G (1977). J Am Chem Soc.

[20] Pignataro S, Foffani A, Grasso F, Cantone B (1965). Z Phys Chem.

[21] Neustetter M, Jabbour Al Maalouf E, Limão-Vieira P, Denifl S (2016). J Chem Phys.

[22] Lengyel J, Kočišek J, Fárník M, Fedor J (2016). J Phys Chem C.

[23] Neustetter M, Mauracher A, Limão-Vieira P, Denifl S (2016). Phys Chem Chem Phys.

[24] Tanaka H, Nakashima N, Yatsuhashi T (2016). J Phys Chem A.

[25] Junk G A, Svec H J (1968). Z Naturforsch, B.

[26] Foffani A, Pignataro S, Cantone B, Grasso F (1965). Z Phys Chem.

[27] Alizadeh E, Ferreira da Silva F, Zappa F, Mauracher A, Probst M, Denifl S, Bacher A, Märk T D, Limão-Vieira P, Scheier P (2008). Int J Mass Spectrom.

[28] Cooks R G, Beynon J H, Caprioli R M (1973). Metastable Ions.

[29] Ferreira da Silva F, Matias C, Almeida D, García G, Ingólfsson O, Flosadóttir H D, Ómarsson B, Ptasinska S, Puschnigg B, Scheier P (2013). J Am Soc Mass Spectrom.

[30] Feketeová L, Postler J, Zavras A, Scheier P, Denifl S, O’Hair R A J (2015). Phys Chem Chem Phys.

